# Comparative Evaluation of Intranasal Ketamine and Midazolam for Premedication in Children Undergoing Elective Surgery: A Prospective Study

**DOI:** 10.7759/cureus.114967

**Published:** 2026-08-22

**Authors:** Vishal P Singh, Kanika Sachdeva, Nigar Bari, Ashutosh Singh

**Affiliations:** 1 Department of Anesthesiology, Shri Guru Ram Rai Institute of Medical and Health Sciences, Dehradun, IND; 2 Department of Emergency Medicine, Shri Guru Ram Rai Institute of Medical and Health Sciences, Dehradun, IND

**Keywords:** general anesthesia, intranasal ketamine, intranasal midazolam, pediatric premedication, sedation

## Abstract

Background: Preoperative anxiety can adversely affect anesthetic induction and perioperative outcomes in children. Intranasal ketamine and midazolam are commonly used noninvasive premedication agents, but evidence comparing their efficacy remains inconsistent. This study compared intranasal ketamine and midazolam in children undergoing elective surgery.

Methods: This prospective comparative study was conducted in the Department of Anesthesiology, Shri Guru Ram Rai Institute of Medical and Health Sciences, Dehradun, India, between August 2023 and July 2025. Seventy-two American Society of Anesthesiologists physical status I children aged three to eight years scheduled for elective surgery under general anesthesia were included. Seventy-two children were assigned alternately, with 36 receiving intranasal ketamine (5 mg/kg) and 36 receiving intranasal midazolam (0.2 mg/kg). Sedation, onset of sedation, child-parent separation, preoperative anxiety, mask acceptance during induction, perioperative hemodynamic parameters, and adverse events were assessed using standardized clinical scales.

Results: Baseline demographic characteristics were comparable between the two groups. The ketamine group demonstrated a significantly shorter onset of sedation than the midazolam group (4.06 ± 0.86 vs. 7.47 ± 1.61 minutes, p < 0.001). Following premedication, Ramsay Sedation Scale scores were significantly higher in the ketamine group, with deep sedation at 20 minutes observed in 19 (52.8%) children compared with nine (25.0%) in the midazolam group (p < 0.001). Child-parent separation was significantly better with ketamine, with excellent separation observed in 30 (83.3%) children compared with two (5.6%) in the midazolam group (p < 0.001). Children receiving ketamine also demonstrated lower preoperative anxiety, with 22 (61.1%) remaining calm and cooperative compared with eight (22.2%) in the midazolam group (p < 0.001), and better mask acceptance during anesthetic induction, with excellent or good acceptance observed in 32 (88.9%) vs. 17 (47.2%) children, respectively (p < 0.001). Heart rate, respiratory rate, systolic blood pressure, and oxygen saturation remained comparable between the groups at most perioperative time points. Ketamine was associated with a lower intraoperative heart rate and higher oxygen saturation after premedication. Mild intraoperative excessive secretions occurred in four (11.1%) children receiving ketamine compared with 0 (0.0%) in the midazolam group, although the difference was not statistically significant (p = 0.115). Postoperative secretions were more frequent with ketamine, occurring in six (16.7%) children compared with 0 (0.0%) in the midazolam group (p = 0.025), whereas postoperative restlessness occurred in seven (19.4%) vs. eight (22.2%) children (p = 0.772). No child in either group experienced postoperative nausea or vomiting, with 0 (0.0%) cases in both groups.

Conclusions: In this prospective comparative study, intranasal ketamine was associated with faster onset and deeper sedation, better child-parent separation, and better mask acceptance than midazolam, with generally stable cardiorespiratory parameters. Postoperative secretions were more frequent with ketamine. Given the alternate allocation, potential unblinding, fixed assessment time, and small single-center sample, these findings are hypothesis-generating and require confirmation in larger randomized multicenter studies.

## Introduction

The administration of premedication in pediatric patients remains a significant perioperative challenge. Young children commonly experience anxiety due to unfamiliar hospital environments, fear of injections and surgery, and separation from their parents [1]. Preoperative anxiety affects approximately 60%-70% of pediatric patients undergoing surgery and is associated with poor cooperation during anesthetic induction, increased perioperative stress responses, emergence delirium, postoperative behavioral disturbances, and delayed recovery [2].

An ideal pediatric premedicant should provide rapid onset of anxiolysis and sedation while preserving cardiorespiratory stability and minimizing adverse effects. Equally important, the route of administration should be painless, child-friendly, and easy to administer with minimal patient cooperation. Although several pharmacological agents, including fentanyl, sufentanil, phencyclidine derivatives, phenobarbital, and ketamine, have been evaluated through oral, intramuscular, intravenous, and intranasal routes, no single drug or route has been shown to be universally superior [3]. In young children, intravenous access is often technically difficult because of small peripheral veins and increased subcutaneous tissue, making noninvasive alternatives particularly attractive [4]. The intranasal route offers several advantages, including rapid systemic absorption through the highly vascular nasal mucosa, avoidance of first-pass metabolism and needle-related discomfort, ease of administration, and high patient acceptance [5].

Midazolam is the most widely used intranasal premedicant because of its anxiolytic, sedative, and amnestic properties. It has a rapid onset of action and is generally effective in facilitating smooth anesthetic induction. However, intranasal midazolam may cause nasal irritation and has been associated with paradoxical reactions, postoperative behavioral changes, cognitive disturbances, and respiratory depression in some patients [6].

Ketamine is another promising intranasal premedication agent with sedative and analgesic properties. As a noncompetitive N-methyl-D-aspartate receptor antagonist, it produces dissociative sedation while preserving spontaneous respiration and protective airway reflexes. Its minimal respiratory depressant effects and favorable cardiovascular profile make it an attractive alternative for pediatric premedication, although increased salivation and prolonged recovery have been reported in some patients [7].

Previous studies comparing intranasal midazolam and ketamine have yielded conflicting results. Lang et al. reported that intranasal midazolam produced more satisfactory sedation with greater hemodynamic stability, faster onset, and shorter recovery than ketamine [2]. In contrast, Gharde et al. demonstrated superior parental separation with intranasal ketamine, suggesting potential advantages during anesthetic induction [8]. These inconsistent findings indicate that the optimal intranasal premedication for children remains uncertain.

The present study aimed to compare the efficacy and safety of intranasal ketamine and intranasal midazolam for preoperative sedation in children undergoing elective surgery. The primary outcome was the level of sedation at the time of parental separation (20 minutes after premedication). Secondary outcomes included onset of sedation, child-parent separation, preoperative anxiety, mask acceptance, perioperative hemodynamic parameters, and perioperative adverse events.

## Materials and methods

Study design and setting

This prospective comparative study was conducted in the Department of Anesthesiology, Shri Guru Ram Rai Institute of Medical and Health Sciences, Patel Nagar, Dehradun, India, over a period of two years from August 2023 to July 2025. The study protocol was approved by the Institutional Ethics Committee, Shri Guru Ram Rai Institute of Medical and Health Sciences, Dehradun (Approval No. SGRR/IEC/23/23; approved on July 15, 2023). Written informed consent was obtained from the parents or legal guardians of all participants before enrolment.

Study participants

Children aged three to eight years, weighing between 10 and 20 kg, and with American Society of Anesthesiologists (ASA) physical status I who were scheduled to undergo elective surgery under general anesthesia were included. Children undergoing emergency surgery or those with a known allergy or contraindication to ketamine or midazolam were excluded from the study.

Sample size

The sample size was calculated based on the difference in satisfactory face mask acceptance reported by Chakraverty et al. [9], where satisfactory mask acceptance was observed in 63.3% of children receiving intranasal ketamine and 30.0% of those receiving intranasal midazolam. Assuming a two-sided alpha error of 0.05 and a study power of 80%, the minimum required sample size was 34 participants per group. To compensate for potential dropouts and exclusions, 36 children were enrolled in each group.

Study procedure

Eligible children were assigned alternately to the ketamine and midazolam groups until the required sample size was achieved. Group A received intranasal ketamine at a dose of 5 mg/kg, whereas Group B received intranasal midazolam at a dose of 0.2 mg/kg. Premedication was administered in the preoperative holding area in the presence of a parent. The calculated dose of the assigned study drug was administered into a single nostril using a 2-mL syringe, with the child in the recumbent position. Ketamine was available at a concentration of 50 mg/mL. For intranasal administration, 2 mL of ketamine was diluted with 8 mL of normal saline to obtain a final concentration of 10 mg/mL and was administered at 5 mg/kg, corresponding to a volume of 0.5 mL/kg. Midazolam was used undiluted at a concentration of 1 mg/mL and administered at 0.2 mg/kg, corresponding to a volume of 0.2 mL/kg. A separate anesthesiologist administered the intranasal medication and was not involved in outcome assessment. Outcome assessors and attending anesthesiologists were not informed of the assigned premedication. However, complete blinding could not be guaranteed because differences in the onset and clinical effects of ketamine and midazolam could potentially allow inadvertent identification of the administered drug.

The assigned drug was instilled into a single nostril using a syringe with the child in the recumbent position. Baseline heart rate, systolic blood pressure (SBP), respiratory rate, and peripheral oxygen saturation (SpO₂) were recorded before administration of the study drug and subsequently at 15-minute intervals until transfer to the operating room.

Sedation was assessed at baseline and after premedication using the Ramsay Sedation Scale (RSS), with sedation additionally assessed at 20 minutes after premedication as the predefined primary outcome assessment [10]. Preoperative anxiety and parental separation were evaluated using a four-point anxiety scale [11], while mask acceptance during induction of anesthesia was assessed using a three-point mask acceptance scale [12]. All assessment scales used in this study were previously published, publicly available clinical assessment tools, and no additional permission was required for their use.

Anesthetic technique

General anesthesia was administered according to a standardized institutional protocol. Induction was performed with sevoflurane in oxygen, following which intravenous access was secured. Glycopyrrolate (0.004 mg/kg) and fentanyl (2 μg/kg) were administered intravenously. Analgesia was supplemented with a diclofenac suppository (2 mg/kg). Endotracheal intubation was facilitated with atracurium (0.5 mg/kg), and anesthesia was maintained with sevoflurane in a mixture of oxygen and 40% nitrous oxide.

Outcome measures

The primary outcome was the level of sedation at parental separation, assessed 20 minutes after premedication. Secondary outcomes included onset of sedation, child-parent separation, preoperative anxiety, mask acceptance during induction, perioperative hemodynamic parameters, and postoperative adverse events. All children were monitored in the recovery room for two hours after surgery. Recorded adverse events included nausea, vomiting, respiratory depression, tachycardia, bradycardia, hypertonia, involuntary movements, shivering, rash, and other drug-related complications.

Statistical analysis

Data were analyzed using IBM Statistical Package for the Social Sciences Statistics for Windows, version 27.0 (IBM Corp., Armonk, NY). Continuous variables were assessed for distributional characteristics before analysis using the Shapiro-Wilk test and graphical assessment where appropriate. Normally distributed continuous variables were compared between groups using the independent-samples t test, with Welch's correction applied when the assumption of equal variances was not satisfied. Ordinal outcomes, including sedation, child-parent separation, preoperative anxiety, and mask acceptance, were analyzed using the Mann-Whitney U test. Categorical variables were compared using Pearson's chi-square test when its assumptions were satisfied. For contingency tables with sparse expected frequencies, Fisher's exact test or the Fisher-Freeman-Halton exact test was used as appropriate. Effect estimates were reported with 95% confidence intervals where applicable. Because multiple exploratory comparisons were performed for perioperative hemodynamic parameters, individual p values were interpreted with caution and no formal multiplicity adjustment was applied. A two-sided p value of <0.05 was considered statistically significant.

## Results

The mean age was comparable between the ketamine and midazolam groups (4.75 ± 1.71 vs. 5.47 ± 1.48 years, p = 0.060). Mean body weight was also comparable between the ketamine and midazolam groups (17.53 ± 2.21 vs. 18.22 ± 1.96 kg, p = 0.163). Both groups had an identical sex distribution, with 19 (52.8%) male and 17 (47.2%) female children in each group (p = 1.000) (Table 1).

**Table 1:** Baseline demographic characteristics of the study participants

Variable	Ketamine (n = 36)	Midazolam (n = 36)	Test statistic	p value
Age (years), mean ± SD	4.75 ± 1.71	5.47 ± 1.48	t(70) = -1.912	0.060
Weight (kg), mean ± SD	17.53 ± 2.21	18.22 ± 1.96	t(70) = -1.411	0.163
Male	19 (52.8)	19 (52.8)	χ²(1) = 0.000	1.000
Female	17 (47.2)	17 (47.2)		

Heart rate was comparable between the ketamine and midazolam groups before premedication (116.78 ± 3.91 vs. 116.14 ± 3.81 beats/minute, p = 0.485), after premedication (117.75 ± 4.76 vs. 117.39 ± 4.91 beats/minute, p = 0.752), and postoperatively (116.36 ± 3.67 vs. 116.78 ± 4.09 beats/minute, p = 0.651). However, the intraoperative heart rate was significantly lower in the ketamine group than in the midazolam group (120.25 ± 3.32 vs. 123.44 ± 3.94 beats/minute, p < 0.001) (Table 2).

**Table 2:** Comparison of perioperative hemodynamic parameters between the study groups SpO₂: peripheral capillary oxygen saturation; SBP: systolic blood pressure

Parameter	Time point	Ketamine (mean ± SD)	Midazolam (mean ± SD)	Test statistic	Mean difference (95% CI)	p value
Heart rate (beats/minute)	Before premedication	116.78 ± 3.91	116.14 ± 3.81	t(70) = 0.702	0.64 (-1.18 to 2.46)	0.485
	After premedication	117.75 ± 4.76	117.39 ± 4.91	t(70) = 0.317	0.36 (-1.91 to 2.63)	0.752
	Intraoperative	120.25 ± 3.32	123.44 ± 3.94	t(70) = -3.723	-3.19 (-4.91 to -1.48)	<0.001
	Postoperative	116.36 ± 3.67	116.78 ± 4.09	t(70) = -0.455	-0.42 (-2.25 to 1.41)	0.651
SpO₂ (%)	Before premedication	98.39 ± 0.49	98.56 ± 0.50	t(70) = -1.416	-0.17 (-0.40 to 0.07)	0.161
	After premedication	99.56 ± 0.50	97.78 ± 1.53	t(42) = 6.611	1.78 (1.24 to 2.31)	<0.001
	Intraoperative	99.94 ± 0.23	99.94 ± 0.23	t(70) = 0.000	0.00 (-0.11 to 0.11)	1.000
	Postoperative	99.97 ± 0.17	99.97 ± 0.17	t(70) = 0.000	0.00 (-0.08 to 0.08)	1.000
Respiratory rate (breaths/minute)	Before premedication	17.53 ± 1.70	17.36 ± 1.81	t(70) = 0.403	0.17 (-0.66 to 0.99)	0.688
	After premedication	17.28 ± 1.86	17.28 ± 1.67	t(70) = 0.000	0.00 (-0.83 to 0.83)	1.000
	Intraoperative	17.67 ± 1.64	17.11 ± 1.35	t(70) = 1.571	0.56 (-0.15 to 1.26)	0.121
	Postoperative	17.06 ± 1.84	17.08 ± 1.76	t(70) = -0.065	-0.03 (-0.87 to 0.82)	0.948
SBP (mmHg)	Before premedication	119.69 ± 5.92	119.42 ± 6.83	t(70) = 0.184	0.28 (-2.73 to 3.28)	0.854
	After premedication	119.78 ± 6.72	120.58 ± 5.64	t(67) = -0.551	-0.81 (-3.72 to 2.11)	0.584
	Intraoperative	118.39 ± 5.69	119.08 ± 5.48	t(70) = -0.527	-0.69 (-3.32 to 1.93)	0.600
	Postoperative	120.31 ± 5.67	119.64 ± 6.17	t(70) = 0.477	0.67 (-2.12 to 3.45)	0.635

SpO₂ was similar between the two groups before premedication (98.39% ± 0.49% vs. 98.56% ± 0.50%, p = 0.161), during the intraoperative period (99.94% ± 0.23% vs. 99.94% ± 0.23%, p = 1.000), and postoperatively (99.97% ± 0.17% vs. 99.97% ± 0.17%, p = 1.000). After premedication, the ketamine group had significantly higher SpO₂ than the midazolam group (99.56% ± 0.50% vs. 97.78% ± 1.53%, p < 0.001) (Table 2).

Respiratory rate remained comparable between the ketamine and midazolam groups at all time points, including before premedication (17.53 ± 1.70 vs. 17.36 ± 1.81 breaths/minute, p = 0.688), after premedication (17.28 ± 1.86 vs. 17.28 ± 1.67 breaths/minute, p = 1.000), intraoperatively (17.67 ± 1.64 vs. 17.11 ± 1.35 breaths/minute, P = 0.121), and postoperatively (17.06 ± 1.84 vs. 17.08 ± 1.76 breaths/minute, p = 0.948) (Table 2).

Similarly, SBP was comparable between the two groups before premedication (119.69 ± 5.92 vs. 119.42 ± 6.83 mmHg, p = 0.854), after premedication (119.78 ± 6.72 vs. 120.58 ± 5.64 mmHg, p = 0.584), intraoperatively (118.39 ± 5.69 vs. 119.08 ± 5.48 mmHg, p = 0.600), and postoperatively (120.31 ± 5.67 vs. 119.64 ± 6.17 mmHg, p = 0.635) (Table 2).

Baseline sedation status was comparable between the ketamine and midazolam groups, with 30 (83.3%) and 27 (75.0%) children being alert, respectively (p = 0.384). Following premedication, RSS scores differed significantly between the groups (Mann-Whitney U = 195.500, p < 0.001), with higher sedation scores observed in the ketamine group. At 20 minutes after premedication, RSS scores were significantly higher in the ketamine group than in the midazolam group (U = 334.000, p < 0.001), with a score of 5 observed in 19 (52.8%) children in the ketamine group compared with nine (25.0%) in the midazolam group. The onset of sedation was also significantly shorter with ketamine than with midazolam (4.06 ± 0.86 vs. 7.47 ± 1.61 minutes; Mann-Whitney U = 35.000, p < 0.001) (Table 3).

**Table 3:** Comparison of sedation characteristics between the study groups

Variable	Category	Ketamine (n = 36)	Midazolam (n = 36)	Test statistic	p value
Baseline sedation score	Alert	30 (83.3)	27 (75.0)	χ²(1) = 0.758	0.384
	Minimal sedation	6 (16.7)	9 (25.0)		
Ramsay Sedation Scale after premedication	1	1 (2.8)	5 (13.9)	U = 195.500	<0.001
	2	4 (11.1)	15 (41.7)		
	3	6 (16.7)	14 (38.9)		
	4	14 (38.9)	2 (5.6)		
	5	11 (30.6)	0 (0.0)		
Sedation after 20 minutes	2	0 (0.0)	9 (25.0)	U = 334.000	<0.001
	3	4 (11.1)	11 (30.6)		
	4	13 (36.1)	7 (19.4)		
	5	19 (52.8)	9 (25.0)		
Onset of sedation (minutes)	Mean ± SD	4.06 ± 0.86	7.47 ± 1.61	U = 35.000	<0.001

Child-parent separation was significantly better in the ketamine group than in the midazolam group (Mann-Whitney U = 126.000, p < 0.001), with 30 (83.3%) children demonstrating excellent separation compared with two (5.6%) children in the midazolam group. Preoperative anxiety scores were significantly lower in the ketamine group than in the midazolam group (Mann-Whitney U = 299.000, p < 0.001), with 22 (61.1%) children remaining calm and cooperative compared with eight (22.2%) in the midazolam group. Similarly, mask acceptance was significantly better in the ketamine group (Mann-Whitney U = 346.000, p < 0.001), with 32 (88.9%) children showing excellent or good acceptance compared with 17 (47.2%) in the midazolam group (Table 4).

**Table 4:** Comparison of perioperative behavioral assessment between the study groups

Variable	Category	Ketamine (n = 36)	Midazolam (n = 36)	Test statistic	p value
Child-parent separation score	Excellent	30 (83.3)	2 (5.6)	U = 126.000	<0.001
	Good	6 (16.7)	28 (77.8)		
	Poor	0 (0.0)	6 (16.7)		
Anxiety score	Calm and cooperative	22 (61.1)	8 (22.2)	U = 299.000	<0.001
	Anxious but could be reassured	11 (30.6)	9 (25.0)		
	Anxious and could not be reassured	3 (8.3)	15 (41.7)		
	Crying or resisting	0 (0.0)	4 (11.1)		
Mask acceptance score	Excellent	16 (44.4)	8 (22.2)	U = 346.000	<0.001
	Good	16 (44.4)	9 (25.0)		
	Fair	4 (11.1)	7 (19.4)		
	Poor	0 (0.0)	12 (33.3)		

Intraoperative excessive secretions were observed in four (11.1%) children in the ketamine group, whereas no child in the midazolam group developed excessive secretions. However, the difference between the two groups was not statistically significant (p = 0.115) (Table 5).

**Table 5:** Comparison of intraoperative findings between the study groups

Variable	Ketamine (n = 36)	Midazolam (n = 36)	Test statistic	p value
No excessive secretions	32 (88.9)	36 (100.0)	Fisher's exact test	0.115
Excessive secretions	4 (11.1)	0 (0.0)		

Postoperative restlessness was comparable between the ketamine and midazolam groups, occurring in 7 (19.4%) and 8 (22.2%) children, respectively (p = 0.772). Postoperative secretions were significantly more frequent in the ketamine group than in the midazolam group, occurring in six (16.7%) children, while no child in the midazolam group developed postoperative secretions (p = 0.025). No child in either group experienced postoperative nausea or vomiting (Table 6).

**Table 6:** Comparison of postoperative complications between the study groups

Variable	Domain	Ketamine (n = 36)	Midazolam (n = 36)	Test statistic	p value
Restlessness	Absent	29 (80.6)	28 (77.8)	χ²(1) = 0.084	0.772
	Present	7 (19.4)	8 (22.2)		
Secretions	Absent	30 (83.3)	36 (100.0)	Fisher's exact test	0.025
	Present	6 (16.7)	0 (0.0)		
Nausea/vomiting	Absent	36 (100.0)	36 (100.0)	-	-
	Present	0 (0.0)	0 (0.0)		

## Discussion

This prospective comparative study evaluated the efficacy and safety of intranasal ketamine and intranasal midazolam as premedication in children undergoing elective surgery. Both groups were comparable with respect to baseline characteristics, including age, sex, and body weight, reducing the likelihood that major demographic differences influenced the observed outcomes. Similar demographic profiles have been reported by Chakraverty et al. [9] and Khatavkar and Bakhshi [13], whereas Marathe and Chhajwani [14] included younger children with lower body weight, which may partly explain differences in clinical responses reported across studies.

Both drugs were associated with satisfactory perioperative hemodynamic stability. Heart rate, respiratory rate, oxygen saturation, and SBP were comparable between the groups at most perioperative time points. The significant differences observed were a lower intraoperative heart rate in the ketamine group and higher oxygen saturation after premedication. Importantly, neither group demonstrated clinically significant respiratory depression or hemodynamic instability. These findings are broadly consistent with studies by Chakraverty et al. [9] and Chouhan et al. [15], who also reported stable perioperative vital parameters following intranasal ketamine and midazolam. Lang et al. [2] observed greater hemodynamic variability with ketamine, although such changes were not clinically significant in their study.

The principal finding of the present study was that ketamine was associated with a faster onset and greater depth of sedation than midazolam. Children receiving ketamine achieved sedation significantly earlier than those receiving midazolam (4.06 ± 0.86 vs. 7.47 ± 1.61 minutes, p < 0.001), and higher RSS scores were observed in the ketamine group. At 20 minutes, a greater proportion of children in the ketamine group had higher sedation scores. These findings indicate an association between intranasal ketamine and more rapid and deeper preoperative sedation in this study population. Similar observations were reported by Chakraverty et al. [9], who demonstrated higher sedation scores with ketamine, and Khoshrang et al. [16], who found improved parental separation with ketamine despite comparable sedation scores. In contrast, Lang et al. [2] and Verma et al. [17] reported faster onset and better sedation with midazolam, highlighting variability among published studies. Differences in patient age, drug dosage, administration techniques, and assessment methods may account for these inconsistent findings.

Behavioral outcomes were more favorable in the ketamine group. Excellent child-parent separation was observed in 83.3% of children receiving ketamine compared with 5.6% in the midazolam group, while preoperative anxiety was significantly lower and mask acceptance was better following ketamine premedication. Nearly 90% of children in the ketamine group demonstrated excellent or good mask acceptance compared with less than half of those receiving midazolam. These findings indicate that ketamine premedication was associated with better perioperative cooperation in the present study. Similar findings have been reported by Chakraverty et al. [9], Marathe and Chhajwani [14], and Chouhan et al. [15], all of whom found smoother parental separation and better procedural acceptance with ketamine. Although Abusinna et al. [18] observed no significant difference between ketamine and midazolam for parental separation, differences among studies may reflect variations in patient characteristics, dosing, administration techniques, and outcome assessment methods.

Both drugs demonstrated an acceptable observed safety profile. No child developed postoperative nausea or vomiting, and postoperative restlessness was comparable between the groups. Mild intraoperative secretions were observed only in the ketamine group, although the difference was not statistically significant. However, postoperative secretions occurred significantly more frequently with ketamine than with midazolam. Despite this finding, the recorded secretions were mild and did not require major intervention. Previous studies by Chakraverty et al. [9], Khatavkar and Bakhshi [13], Chouhan et al. [15], and Verma et al. [17] similarly reported a low incidence of adverse effects with both agents. Overall, the present findings suggest an association between intranasal ketamine at a dose of 5 mg/kg and faster and deeper sedation, better child-parent separation, lower preoperative anxiety, and better mask acceptance compared with intranasal midazolam at a dose of 0.2 mg/kg, with postoperative secretions occurring more frequently with ketamine.

The present study has several strengths. It prospectively compared two commonly used intranasal premedication agents using standardized anesthetic protocols and structured assessment scales for sedation, child-parent separation, anxiety, and mask acceptance. Perioperative hemodynamic parameters and adverse events were systematically monitored, providing an assessment of both efficacy-related and safety outcomes. The use of a uniform study protocol and equal sample sizes facilitated standardized assessment across the two groups.

However, the study has certain limitations. It was conducted at a single tertiary care center with a relatively small sample size and included only ASA physical status I children aged three to eight years undergoing elective surgery; therefore, the findings may not be generalizable to younger infants, older children, emergency procedures, or children with significant comorbidities. Postoperative recovery characteristics, long-term behavioral outcomes, and parental satisfaction were not evaluated. Alternate allocation rather than true randomization was used for group assignment. Although this approach provided equal group sizes through a predefined allocation sequence, it does not provide the protection against selection bias afforded by randomization and may have affected internal validity. Sedation and behavioral outcomes were ordinal and were therefore analyzed using nonparametric methods, while exact procedures were used for contingency tables with sparse expected frequencies. Multiple between-group comparisons were performed for perioperative hemodynamic parameters across several time points without formal adjustment for multiplicity; these analyses were considered exploratory, and isolated statistically significant findings should therefore be interpreted cautiously. In addition, separate comparisons at different perioperative time points did not account for within-participant correlation over time. Finally, complete blinding could not be guaranteed because differences in the onset and clinical effects of ketamine and midazolam could potentially allow treatment allocation to be inferred. Larger multicenter studies with adequately powered samples, true randomization, longer follow-up, and assessment of postoperative recovery and parental satisfaction are warranted to validate these findings across a broader range of pediatric populations.

## Conclusions

In this prospective comparative study, intranasal ketamine was associated with faster onset and deeper sedation, better child-parent separation, and better mask acceptance than intranasal midazolam in children undergoing elective surgery. Perioperative cardiorespiratory parameters remained generally stable in both groups, although secretions were more frequent with ketamine. These findings should be interpreted cautiously because of the alternate allocation design, potential for inadvertent unblinding, fixed time point of outcome assessment, and relatively small single center sample. The findings are hypothesis generating and should not be interpreted as definitive evidence of comparative superiority. Larger randomized multicenter studies with appropriately timed repeated assessments are warranted to confirm these observations and establish the comparative effectiveness and safety of intranasal ketamine and midazolam.
